# Effective Mechanical Properties and Thickness Determination of Boron Nitride Nanosheets Using Molecular Dynamics Simulation

**DOI:** 10.3390/nano8070546

**Published:** 2018-07-19

**Authors:** Venkatesh Vijayaraghavan, Liangchi Zhang

**Affiliations:** Laboratory for Precision and Nano Processing Technologies, School of Mechanical and Manufacturing Engineering, The University of New South Wales, Sydney, NSW 2052, Australia; venkatesh.vijayaraghavan@unsw.edu.au

**Keywords:** boron nitride nanosheet, molecular dynamics, thickness, mechanical strength, vacancy defects

## Abstract

Research in boron nitride nanosheets (BNNS) has evoked significant interest in the field of nano-electronics, nanoelectromechanical (NEMS) devices, and nanocomposites due to its excellent physical and chemical properties. Despite this, there has been no reliable data on the effective mechanical properties of BNNS, with the literature reporting a wide scatter of strength data for the same material. To address this challenge, this article presents a comprehensive analysis on the effect of vital factors which can result in variations of the effective mechanical properties of BNNS. Additionally, the article also presents the computation of the correct wall thickness of BNNS from elastic theory equations, which is an important descriptor for any research to determine the mechanical properties of BNNS. It was predicted that the correct thickness of BNNS should be 0.106 nm and the effective Young’s modulus to be 2.75 TPa. It is anticipated that the findings from this study could provide valuable insights on the true mechanical properties of BNNS that could assist in the design and development of efficient BN-based NEMS devices, nanosensors, and nanocomposites.

## 1. Introduction

Research on boron nitride nanosheets (BNNS) has evoked great prominence in recent days owing to its unique physical and electronic properties [1,2]. BNNS is the best example of a 2D single-layer compound nanomaterial, consisting of boron and nitrogen atoms in equal numbers in a hexagonal lattice arrangement. While the mechanical strength of BNNS is not as high compared to that of its elemental counterpart—graphene [3], it possesses better thermal and oxidation resistance [4]. This makes BNNS an attractive alternative to graphene for applications under extreme conditions, such as nanocomposites [5,6], nanoelectronics [7,8], and nanoelectromechanical (NEMS) devices [9,10]. This creates a major initiative in investigating the mechanical strength of BNNS, which will provide valuable information for the design of next-generation BNNS-based nano-devices and components.

Previous studies investigating the mechanics of BNNS by experiments mainly focused on reporting the Young’s modulus of BNNS. The reported Young’s modulus of BNNS was scattered and was found to be dependent on the method of fabrication of the BNNS. For instance, Falin et al. [1] and Bosak et al. [11] and devised the fabrication of BNNS by exfoliation from BN crystals and found the Young’s modulus of BNNS to be 0.865 and 0.811 TPa, respectively. However, Song et al. [12] predicted that the BNNS fabricated by the chemical vapour deposition (CVD) process, yielded a Young’s modulus of only about 0.334 TPa. The low strength was attributed to the inherent defects and grain boundaries resulting from the CVD process. Kim et al. [13], on the other hand, adopted the CVD process using an iron foil with a borazine precursor to synthesize high-quality BNNS. They reported that the Young’s modulus of BNNS can be substantially higher for the case of BN with little or no inherent defects and obtained a Young’s modulus of 1.16 ± 0.1 TPa. A similar observation was also reported for the case of boron nitride nanotubes (BNNTs) by Chopra and Zettl [14] who devised a water cooled arc for synthesizing pure BNNTs. They found that the Young’s modulus of BNNS is 1.22 ± 0.24 TPa. Suryavanshi et al. [15] measured the Young’s modulus of 18 different BNNTs with varying lengths and diameters and also reported a wide scatter of the Young’s modulus, varying from 0.505–1.031 TPa. These studies also adopted varying measurement techniques, such as atomic force microscopy (AFM), transmission electron microscopy (TEM), inelastic X-ray scattering (IXS) technique, etc., to name a few. The measurement errors in these techniques might have also contributed to the diverse range of strength data of BNNS available in the literature. A summary of the mechanical strength of BNNS as reported from the abovementioned experimental studies are presented in Table 1.

It is evident from the above experimental studies that the variation in the Young’s modulus and the mechanical strength of BNNS can be influenced by many factors. Computational modelling has emerged as an effective means of studying the influence of various parameters, such as defects, geometry, and lattice orientation on the strength data of BNNS. Molecular dynamics (MD) studies [16,17,18] showed that the Young’s modulus of BNNS is highly sensitive to defects in BNNS lattice. Similar conclusions were also obtained from density functional theory (DFT) analysis by Wang et al. [19]. Another advantage of deploying computational model is that the lattice parameters or geometry of BNNS can be easily modified and the resulting mechanical strength can be estimated. For instance, Le [20] adopted a molecular mechanics (MM) model for BNNS undergoing tensile loading, and found that BNNS loaded in armchair direction exhibits lower tensile strength. This observation was also confirmed by MD simulation results of Mortazavi and Rémond [21] and DFT analysis by Wu et al. [22]. In addition to defects and lattice orientation, the strength of BNNS was also reported to be strongly influenced by temperature. Adopting a quasi-harmonic approximation (QHA) model, Mirnezhad et al. [23] showed that the Young’s modulus of BNNS is highly sensitive to temperature, reaching a stable value at elevated temperature. Other computational approaches, such as hybrid Tersoff-Brenner (T-B) [24] and continuum-lattice (C-L) [25] models, also reported similar results. Some computational studies also analysed the strength variation of boron nitride nanotubes (BNNTs) [26] and their strength comparison with carbon nanotubes or graphene [27,28]. Most of these studies reported the Young’s modulus and mechanical strength of BNNS by considering the wall thickness of BNNS to be around 3.3 to 3.4 Å. Some studies also reported thickness-independent mechanical strength descriptors, such as the axial stiffness and bending stiffness from conventional modelling techniques, such as MD simulations [29,30] or other techniques, such as atomistic-finite element modelling (FEM) [31] or the discrete media homogenization (DMH) technique [32]. A consolidation of all mechanical properties of BNNS investigated by various computational approaches is presented in Table 2.

From the literature studies presented above, it is possible to map the effect of individual factors on the mechanical strength of BNNS. Table 3 presents the effect of various factors which can influence the mechanical characteristics of BNNS. This offers a quick glance of the dominant factors which results in the variation of the reported strength data of BNNS. It is also possible to determine the knowledge gaps on the existing studies on the mechanics of BNNS. For instance, while it is evident that the strength of BNNS is adversely affected by the presence of defects and increasing temperature, superior mechanical properties can be obtained by orienting the BNNS along a zigzag direction. While the effect of individual factors, such as defects and temperature, has been well documented, none of the abovementioned studies focused on analysing the effect of BNNS geometry, the position of defects, and the combined effect of two or more factors in influencing the strength data of BNNS. Additionally, almost all of the existing studies in the literature reported the mechanical strength and Young’s modulus of BNNS by assuming the thickness of BNNS to be 3.3 to 3.4 Å, which is the inter-layer separation distance of graphene. This assumption is not true given that the BNNS exhibits a discrete hexagonal lattice arrangement of atoms and, hence, its wall thickness is not well defined. Estimating the correct wall thickness is very crucial to determining the effective Young’s modulus and mechanical properties of BNNS at the nanoscale. Assuming an incorrect wall thickness leads to a scatter of the Young’s modulus and mechanical strength data of BNNS, this leads to a critical knowledge gap on exploiting these nanomaterials for high-strength applications.

Motivated by the above research questions, this article aims to provide a comprehensive analysis on the mechanics of BNNS under tensile loading conditions. To this end, the critical factors which can result in a variation of strength data of BNNS are identified and the extent to which the strength data is varied is determined first. Additionally, this article also addresses the need to compute the correct wall thickness of BNNS, and thereby its effective Young’s modulus and mechanical strength at the nanoscale. The correct wall thickness of the BNNS is estimated by adopting the Vodenitcharova-Zhang [40] and Wang-Zhang [41] criteria. Once the correct wall thickness is determined, the effective Young’s modulus and the mechanical strength of BNNS is then calculated and presented.

## 2. Computational Model

This article focuses on the mechanics of a single-layer BNNS using MD simulation. The simulations are performed on the large scale atomic/molecular massively parallel simulator (LAMMPS) package of March 2017 version, developed by Sandia National Laboratories, Livermore, CA, USA [42]. The modified Tersoff potential [43,44] with optimized parameters defined by Kinaci et al. [45] is used to describe the interactions between the boron and nitrogen atoms of BNNS. The Tersoff potential, with its precise parameters, has the ability to accurately match the experimental results with density functional theory calculations, while also ensuring the computational efficiency for large-scale atomic systems [46]. In addition, the Tersoff potential has also been successfully used in previous studies on computational modelling of BNNS [21,47,48,49]. The complete details of this potential function with associated parameters can be found in [18].

**Figure 1:**
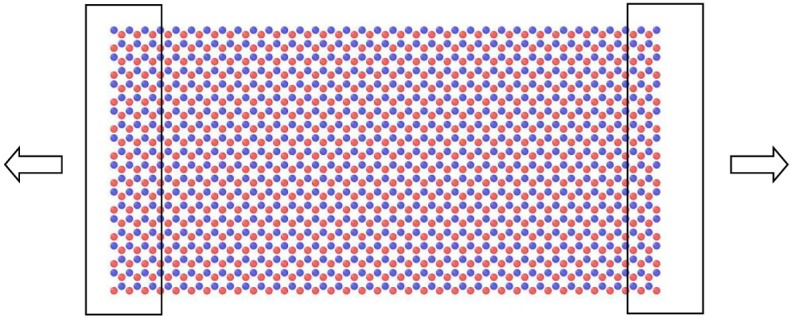
Simulation of BNNS under tensile loading. The atoms enclosed inside the black rectangle at either ends of BNNS is subjected to tensile loading. The loading direction is indicated by the arrows. Atoms depicted in ochre are boron and atoms depicted in blue are nitrogen.

The studies described in this paper analyses the effect of geometry, loading direction, defects, and temperature on the mechanical strength of BNNS. The effect of geometry is considered by suitably modifying the aspect ratio (ratio of length to the width) of the BNNS in zigzag and armchair directions. The effect of concentration and position of vacancy defects is studied by constructing various concentrations axial or transverse defects along the direction of loading of the BNNS. The temperature factor is investigated by subjecting the BNNS structure to tensile loading at 300, 600, and 900 K. At the beginning, the BNNS is equilibrated at the specific temperature, after which the boundary atoms of the BNNS are fixed and subjected to constant outward displacement to simulate tension (Figure 1). The BNNS is again equilibrated at every 1000 time steps to relax the structure, after which the readings are recorded and the procedure is repeated until the BNNS fails under tension.

## 3. Results and Discussion

### 3.1. Validation of the Simulation Model

**Figure 2:**
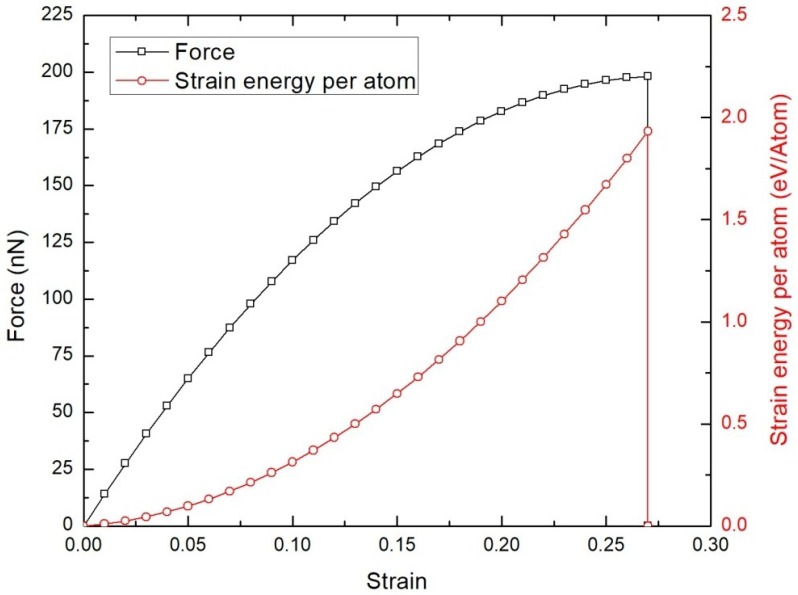
Force and strain energy graph of BNNS under tensile loading at 300 K.

The simulation model adopted in the present study is validated by considering an approximate square shaped BNNS of dimension 62.38 Å × 60.27 Å consisting of 700 boron and nitrogen atoms. The BNNS is loaded in tension along the zigzag direction as depicted in Figure 1 at 300 K. The plot of the force and strain energy per atom measured against the tensile strain is illustrated in Figure 2. The plot shows that the tensile force varies almost linearly with strain, *ε*, until *ε* = 0.05. After this, the force shows a parabolic variation until it reaches a maximum value of 198.14 nN before undergoing failure as indicated by the spontaneous drop of strain energy. The observed tensile strain of *ε* = 0.27 from the present MD simulation is in good agreement with the finite element prediction value of 0.257 by Le and Nguyen [39] who adopted a square-shaped model of BNNS. The computed axial stiffness of the BNNS (defined as twice the coefficient of the second degree term of the strain energy polynomial curve) is 277.4 J/m^2^ which is comparable with the ab initio prediction of 271 J/m^2^ [35] and MD prediction of 267 J/m^2^ [29]. Hence, the above confirmation study validates the accuracy of the simulation model adopted in the study.

### 3.2. Effect of Geometry and Tensile Loading Direction

**Figure 3:**
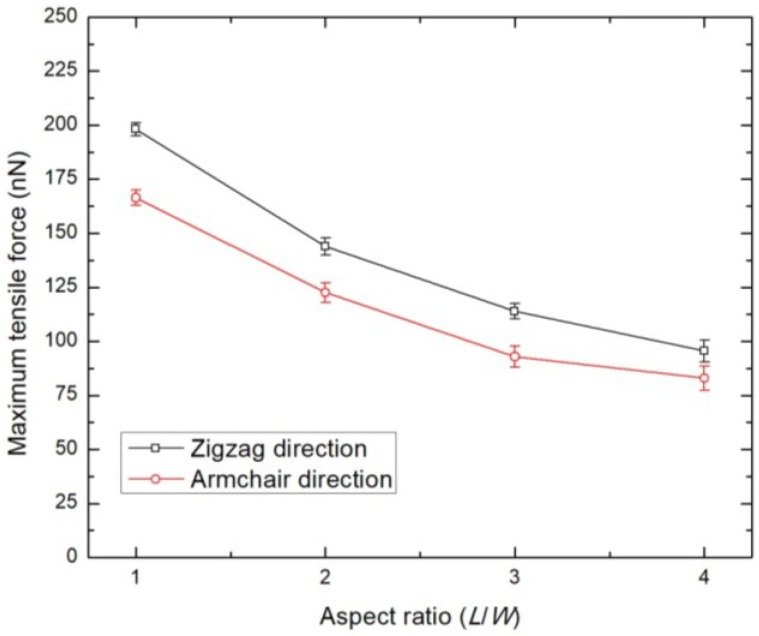
Tensile loading characteristics of single layer BNNS of varying aspect ratios at 300 K.

The effect of geometry and tensile loading direction on the mechanical characteristics of BNNS is described in this section. It is useful to note that previous studies have modelled BNNS with varying geometry, such as rectangular [18,21], square shaped [39], or even as a circle [50], and have reported varying data of tensile strength of single-layer BNNS. The strength data of BNNS should be ideally investigated by standardizing the variation in geometry. This is accomplished in this work by varying the aspect ratio (ratio of length, *L* to the width, *W*) of BNNS, while maintaining almost the same number of boron and nitrogen atoms. The effect of the loading direction is considered by tensile loading of BNNS along either armchair or zigzag directions. The geometry and atomic configuration of armchair and zigzag BNNS considered in the study is presented in Table 4 and Table 5, respectively. The plot of the maximum tensile force for armchair and zigzag BNNS as a function of the aspect ratio at 300 K is shown in Figure 3. The plot shows that the BNNS exhibits superior tensile strength when loaded in the zigzag direction, which is consistent with the previous literatures. Additionally, the maximum tensile force of BNNS decreases with the increasing aspect ratio. This is because a small aspect ratio BNNS is wider than a larger aspect ratio BNNS. In the present study, as there is minor variation in the atom numbers of BNNS across aspect ratios, a wider BNNS has more atomic bonds which can resist the tensile loading. Furthermore, the loading characteristics tend to stabilize with the increasing aspect ratio of the BNNS. Hence, the mechanical strength of the BNNS can be effectively modified by varying the system geometry.

**Figure 4:**
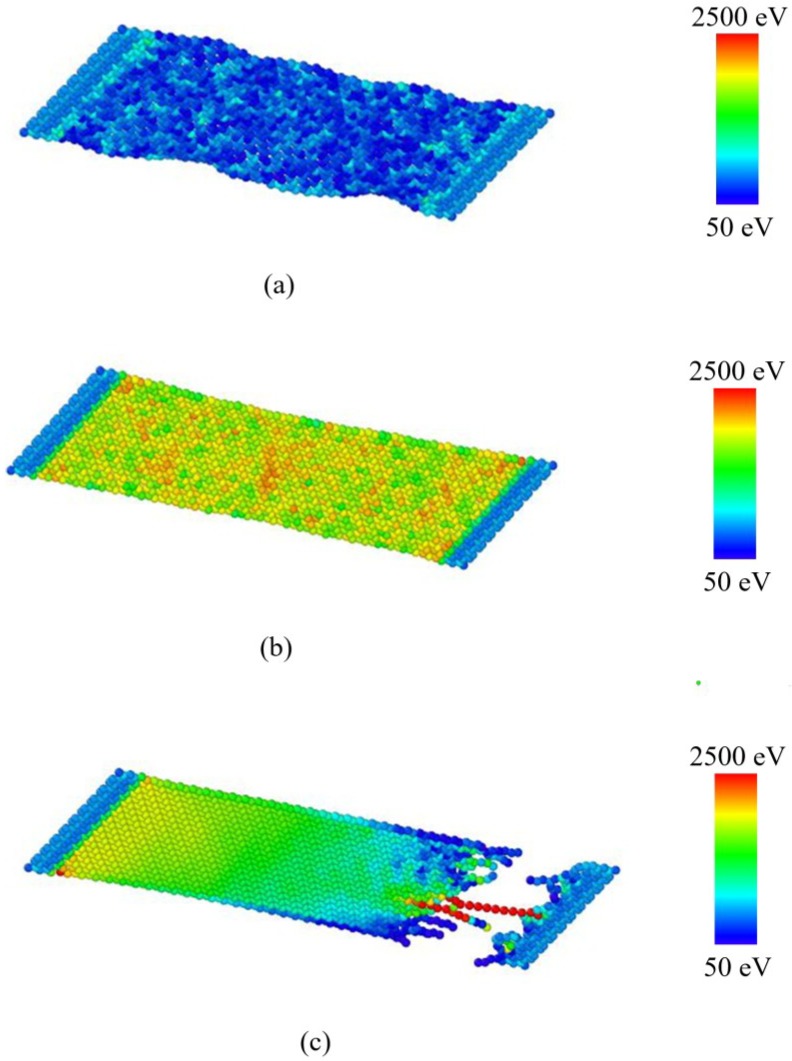
Tensile loading stages of single-layer BNNS at 300 K at (**a**) *ɛ* = 0.0; (**b**) *ɛ* = 0.12; and (**c**) *ɛ* = 0.25.

The snapshots of the single layer BNNS of aspect ratio 2.0 undergoing tensile loading at 300 K is depicted in Figure 4. The initial equilibration at 300 K results in some wrinkles on the BNNS structure. The application of tensile displacement results in elongation and increase in strain energy of the atoms, which results in tensile failure of the BNNS sheet marked by fracture and segmentation.

### 3.3. Effect of the Concentration and Position of Vacancy Defect

**Figure 5:**
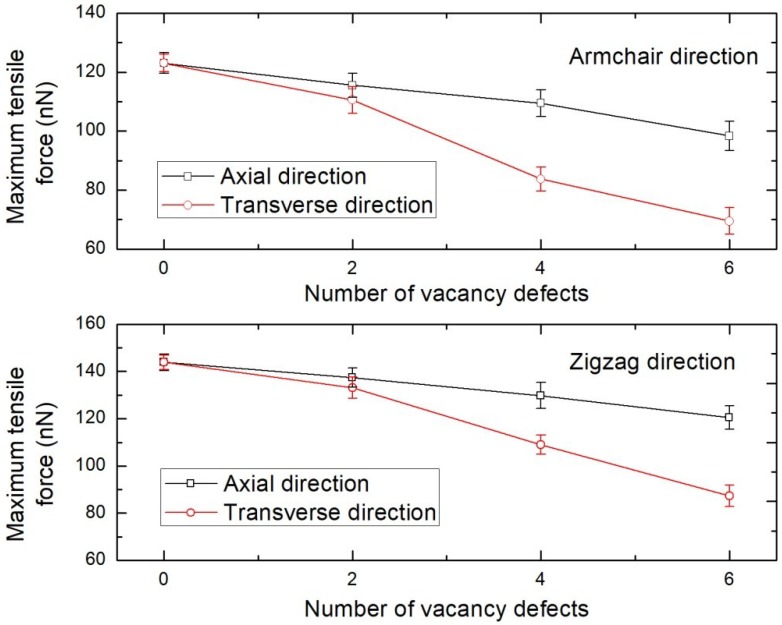
Tensile loading characteristics of single layer BNNS with vacancy defects along the axial and transverse directions at 300 K.

The influence of vacancy defects on the tensile loading characteristics of BNNS is investigated by considering a BNNS sheet of aspect ratio 2.0, loaded in armchair and zigzag directions at 300 K. The vacancy defect is constructing by removing a set of covalently bonded boron and nitrogen atoms from the BNNS lattice. Figure 5 shows that increasing the concentration of defects deteriorates the mechanical strength of BNNS. It is, however, intriguing to note that the extent of the influence exerted by the defects has strong dependency on their position or placement in the BNNS. For instance, the maximum tensile force for an armchair BNNS drops by over 43% when the vacancy defect concentration along the transverse direction (i.e., perpendicular to direction of loading) is increased to 6. However, the corresponding reduction in the maximum tensile force when the defects are located in the axial direction is only about 20%. This is an important characteristic which should be taken note of while synthesizing BNNS for composite loading or high-strength applications. Hence, the vacancy defects along the transverse direction must be minimized as much as possible to avoid the rapid deterioration in the mechanical properties of BNNS.

### 3.4. Effect of Temperature and Vacancy Defects

**Figure 6:**
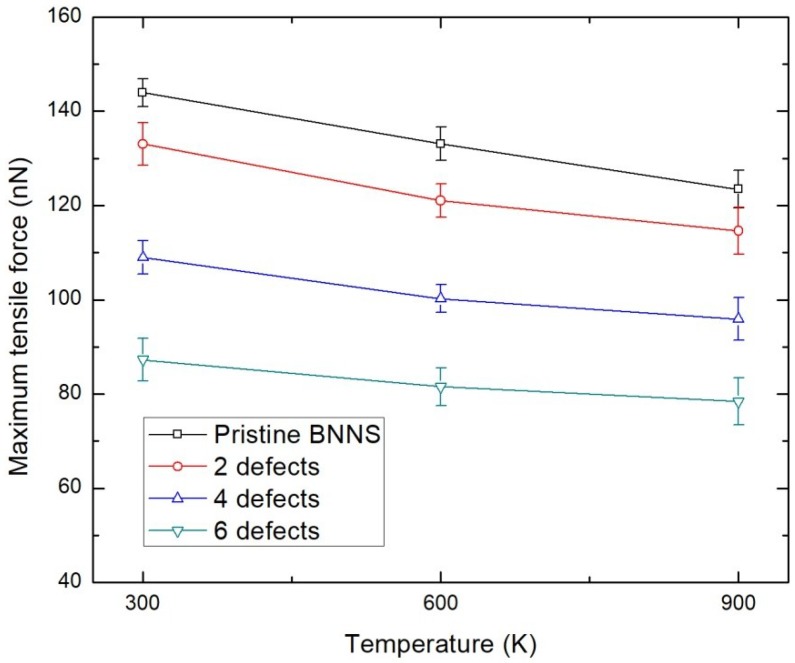
Tensile loading characteristics of BNNS at various temperatures with vacancy defects.

The previous studies presented in the introduction indicated that the strength of BNNS is strongly affected by temperature and the existence of defects. However, the strength of defective BNNS has not been tested in elevated temperatures, or vice versa, which will provide a comprehensive understanding of the interaction of these two dominant factors. Figure 6 depicts the variation in tensile loading characteristics of BNNS with increasing defect concentration at various temperatures. It can be witnessed that the increase in temperature decreases the maximum tensile force of the BNNS, which is attributed to the increase in the thermal stress on B–N bonds. However, it is also interesting to note that introducing the vacancy defects in the BNNS structure seems to mitigate the weakening effect of BNNS caused due to the rise in temperature (Table 6). For instance, the reduction in the maximum tensile force of a pristine BNNS when the temperature is increased from 300 to 900 K is 14.25%, while the corresponding drop for a BNNS with six vacancy defects is found to be lowered to 10.16%. The loss of B–N bonds formed in BNNS due to the presence of vacancy defects enhances the mobility of the atoms at higher temperature which results in the lowering of associated thermal stress in the BNNS structure. This observation is also consistent with the mechanics of the graphene sheet [51] and buckling of CNTs [52] analysed at higher temperatures. Hence, BNNS with a higher concentration of vacancy defects resists the drop in tensile loading characteristics due to temperature increases. This could be an important factor which could be exploited while synthesizing BNNS for high strength applications under elevated temperatures. Another useful feature which can be deduced from this study is that the drop in loading characteristics of BNNS due to temperature variation is not as pronounced when compared to that of graphene as reported by authors’ previous study [51]. Hence, this investigation supports the fact that BNNS exhibits better thermal stability and can be used for fabricating temperature-resistant nanoscale devices and nanocomposites.

## 4. Determination of Thickness and the Young’s Modulus of BNNS

Almost all of the previous studies on computational modelling of BNNS have assumed the thickness to be 3.4 Å—the inter-layer separation between two graphene sheets. This yields the Young’s modulus to be about 0.6–0.9 TPa. The application of the same thickness to estimate the mechanical characteristics of BNNS is questionable since the effective thickness of graphene itself was computed to be between 0.06 to 0.1 nm [41,53].

To overcome this hurdle, the effective thickness of BNNS is determined in this work based on the well-established Vodenitcharova-Zhang [40] and Wang-Zhang [41] criteria. In so doing, the axial stiffness, *K*, and bending stiffness, *D*, are firstly determined without using *E* and *h* values. In the atomistic simulation of BNNS, the axial stiffness *K* is defined as [41]:

(1) $$\left\{ \begin{array}{l} K=\frac{1}{A}\cdot \frac{\partial^{2}W_{a}}{\partial \varepsilon^{2}}\\ W=a_{0}+a_{1}\varepsilon +a_{2}\varepsilon^{2}+a_{3}\varepsilon^{3}+\cdots \end{array}\right.$$

where *W_a_* is the strain energy of the BNNS structure under axial loading, *A* is the surface area of the BNNS, *a_j_* (*j* = 0,1,2,3,…) is the coefficient of the fitted polynomial of *W_a_* in terms of strain, and *ε* derived from the strain energy-strain plot.

**Figure 7:**
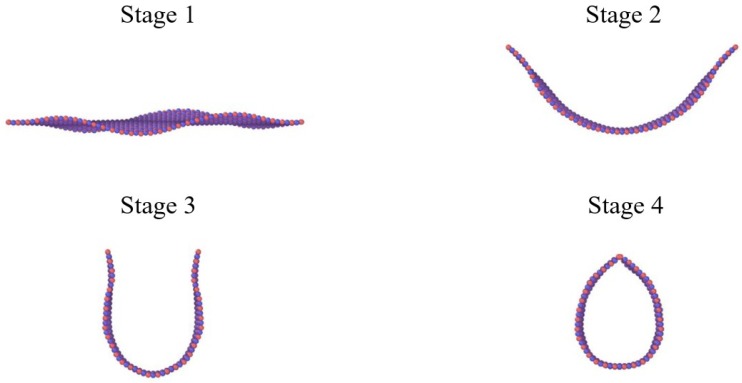
Stages of bending in BNNS to form a BNNT.

The bending stiffness, *D,* of BNNS is determined by the energy required in rolling up the BNNS surface to form a BNNT (see Figure 7). *D* is defined mathematically as [41]:

(2) $$\left\{ \begin{array}{l} D=\frac{1}{A}\cdot \frac{\partial^{2}W_{b}}{\partial \kappa^{2}}\\ W_{b}=b_{0}+b_{1}\kappa +b_{2}\kappa^{2}+b_{3}\kappa^{3}+\cdots \end{array}\right.$$

where *W_b_* is the energy of the BNNS structure during bending process to form a BNNT, *b_j_* (*j* = 0,1,2,3,…) is the coefficient of the fitted polynomial of *W_b_* in terms of curvature, and *κ* is derived from the energy-curvature plot.

Based on elastic theory, the *K* and *D* values are defined in terms of *E* and *h* as [41]:

(3) $$K=\frac{Eh}{1-\upsilon^{2}}\approx Eh$$

(4) $$D=\frac{Eh^{3}}{12(1-\upsilon^{2})}\approx \frac{Eh^{3}}{12}$$

The above equations are then solved to determine the unique values of *E* and *h*, which is sufficient to satisfy the axial stiffness and bending stiffness of BNNS. In addition, it is also necessary for the resultant thickness to be smaller than that of the atomic diameter, since the cross-section of the sheet only consists of discrete atoms connected by bonds, as opposed to a continuous wall of atoms.

**Figure 8:**
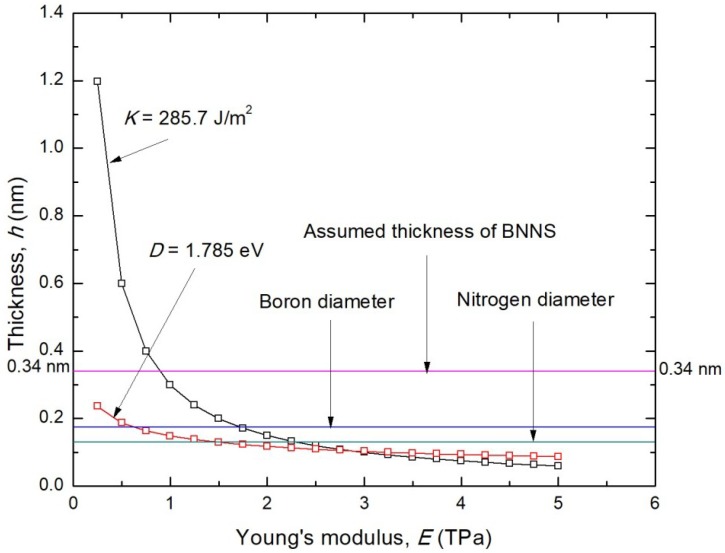
Determination of the correct thickness and Young’s modulus of BNNS from the intersection of axial stiffness and bending stiffness curves on the *E*-*h* coordinate plane.

The variation of thickness with the Young’s modulus is plotted on a single *E*-*h* coordinate plane using Equations (3) and (4), as shown in Figure 8. In the present study, the average *K* and *D* values of BNNS at various aspect ratios were obtained as 285.7 J/m^2^ and 1.785 eV, respectively. These values are in good agreement with the *K* and *D* values computed from various numerical approaches, as illustrated in Table 1. From Figure 7, the correct thickness of BNNS is determined by the intersection of the *K* and *D* curves, while also satisfying the Vodenitcharova-Zhang necessary criterion [40]. Hence, the correct effective thickness of BNNS is *h* ≈ 0.106 nm and the Young’s modulus ≈ 2.75 TPa.

The resulting Young’s modulus is higher than that of the previously reported estimates using computer simulation studies. Hence, the following checks can be conducted to confirm the validity of the computed Young’s modulus:

(1) The correct wall thickness for graphene was estimated to be about 0.10 nm [41,54]. Since BNNS is morphologically similar to the graphene sheet, the BNNS thickness of 0.106 nm is closely comparable to the thickness of the graphene sheet.
(2) For a thickness of 3.4 Å, the Young’s modulus of the BNNS reported by computational studies is lower than that of graphene, and should be valid regardless of any thickness considered. As the computed modulus of BNNS (2.75 TPa) is lower than the correct modulus of graphene, which is reported to be 3.4–3.5 TPa [41,55], the above findings can be validated.

Using the effective thickness of 0.106 nm and the maximum tensile force values reported for the armchair and zigzag BNNS in Section 3.2, the mechanical strength of the BNNS with varying aspect ratios are computed and presented in Table 7.

## 5. Conclusions

Mechanical loading characteristics of BNNS under tensile loading conditions have been comprehensively analysed in this work. A detailed literature review has been conducted to consolidate the effect of various factors which can influence the quantification of the mechanical properties of BNNS. Based on the literature consolidation, it is identified that the influence of geometry, defect position, and the combination of defects and temperature on the property characterization of BNNS must be investigated. The variation in the system geometry was standardized across BNNS by maintaining almost a similar number of atoms in the sheet. Through this, it was found that a smaller aspect ratio of BNNS exhibits better tensile loading characteristics. Furthermore, while increasing the defect concentration, itself, can deteriorate the mechanical strength of BNNS, the extent of the reduction was found to have a strong dependency on the position or placement of the defects. The study also revealed interesting phenomena that these vacancy defects can control the decline in tensile loading characteristics of BNNS due to elevated temperatures. Hence, it would be favourable to include vacancy defects in BNNS for high-temperature applications, albeit placing the defects along the direction of loading of BNNS. Finally, the effective Young’s modulus of the BNNS is also estimated by computing the correct wall thickness based on elastic theory equations. It is anticipated that the comprehensive analysis presented in this work will provide valuable information for the fabrication of BNNS-based NEMS, nanoscale devices, and nanocomposites.

## Figures and Tables

**Table 1:** Mechanical properties of BNNS and BNNTs determined by experiments.

Experimental Method	Young’s Modulus (TPa)
Nanoindentation measurement of few layer BNNS exfoliated from single crystal BN [1].	0.865 ± 0.073
IXS of BNNS crystal synthesized from Ba-B-N catalyst system under high temperature and pressure [11].	0.811
Nanoindentation measurement on defective BNNS synthesized by CVD from bulk BN crystal [12].	0.334 ± 0.024
AFM measurement on high quality BNNS synthesized from borazine precursor using CVD process [13].	1.16 ± 0.1
Thermal assisted vibration of cantilevered BNNT observed using TEM [14].	1.22 ± 0.24
Electric-field-induced technique to apply sinusoidal signal which induces vibration in BNNT [15].	0.505–1.031

**Table 2:** Mechanical properties of BNNS and BNNTs by computational modelling.

Technique	Temperature (K)	Young’s Modulus (TPa)	Axial Stiffness (TPa nm)	Bending Stiffness (eV)
Tersoff potential [16]	300	0.930	NA	NA
Tersoff potential [17]	NA	0.730–0.890	0.248–0.292	NA
Tersoff potential [18]	0–2000	0.398–0.720	NA	NA
DFT calculation [19]	NA	NA	0.293–0.311	NA
Mechanics model [20]	0	NA	0.332	NA
Tersoff potential [21]	300	0.800–0.850	0.264–0.280	NA
DFT calculation [22]	NA	0.760–1.055	NA	0.95
DFT-QHA model [23]	0–1000	NA	0.278–0.283	NA
T-B potential [24]	300	0.881	NA	NA
Continuum model [25]	NA	0.900–1.000	NA	NA
Tight binding [26]	NA	NA	0.284–0.310	NA
MM-DFT model [27]	NA	0.83	0.282	1.74
DFT calculation [28]	NA	0.700–0.830	NA	NA
Tersoff potential [29]	0	NA	0.267	NA
Tersoff-like model [30]	300	NA	NA	1.5–1.7
Atomistic-FEM [31]	NA	NA	0.240–0.315	NA
DMH technique [32]	NA	NA	0.267	NA
Tersoff potential [33]	NA	0.295–0.695	NA	0.22–0.56
MM model [34]	NA	NA	0.260–0.269	NA
Ab initio [35]	NA	NA	0.271	1.29
DFT calculation [36]	NA	NA	0.279	NA
Modified T-B [37]	NA	0.982–1.113	NA	NA
Tersoff potential [38]	300	0.716	NA	NA
Tersoff potential [39]	0	0.749–0.770	0.248–0.258	NA

**Table 3:** Relationship mapping of the effect of various factors on the mechanical strength of BNNS.

Factors	Tensile Strength of BNNS
Temperature	Decreases
Defect concentration	Decreases
Geometry	Unknown
Defect position	Unknown
Defects and Temperature	Unknown
Loading direction	Superior in zigzag direction

**Table 4:** The atomic configuration of single layer BNNS loaded in the zigzag direction.

Aspect Ratio (*L*/*W*)	BNNS Dimensions (*L* × *W*)	Total Number of Atoms
1.0	62.38 Å × 60.27 Å	1408
2.0	89.11 Å × 42.63 Å	1420
3.0	104.39 Å × 33.81 Å	1328
4.0	120.94 Å × 29.40 Å	1344

**Table 5:** The atomic configuration of single layer BNNS loaded in the armchair direction.

Aspect Ratio (*L*/*W*)	BNNS Dimensions (*L* × *W*)	Total Number of Atoms
1.0	62.48 Å × 61.11 Å	1450
2.0	86.73 Å × 43.28 Å	1400
3.0	104.37 Å × 33.10 Å	1344
4.0	119.81 Å × 30.55 Å	1374

**Table 6:** Percentage reduction of the maximum tensile force of BNNS with defects when temperature is increased from 300 to 900 K.

Number of Defects	Reduction of Maximum Tensile Force (%)
0	14.25
2	13.83
4	11.97
6	10.16

**Table 7:** Effective mechanical strength of armchair and zigzag BNNS at various aspect ratios.

Armchair BNNS		Zigzag BNNS	
Aspect Ratio	Mechanical Strength (GPa)	Aspect Ratio	Mechanical Strength (GPa)
1.0	254.31	1.0	302.75
2.0	266.82	2.0	318.51
3.0	259.33	3.0	317.70
4.0	266.21	4.0	306.67

## References

[1] Falin A., Cai Q., Santos E.J.G., Scullion D., Qian D., Zhang R., Yang Z., Huang S., Watanabe K., Taniguchi T. (2017). Mechanical properties of atomically thin boron nitride and the role of interlayer interactions. Nat. Commun..

[2] Zeng H., Zhi C., Zhang Z., Wei X., Wang X., Guo W., Bando Y., Golberg D. (2010). “White graphenes”: Boron nitride nanoribbons via boron nitride nanotube unwrapping. Nano Lett..

[3] Zhang J., Wang C. (2016). Mechanical properties of hybrid boron nitride-carbon nanotubes. J. Phys. D Appl. Phys..

[4] Golberg D., Bando Y., Huang Y., Terao T., Mitome M., Tang C., Zhi C. (2010). Boron nitride nanotubes and nanosheets. ACS Nano.

[5] Isarn I., Ramis X., Ferrando F., Serra A. (2018). Thermoconductive thermosetting composites based on boron nitride fillers and thiol-epoxy matrices. Polymers.

[6] Yu J., Zhao W., Wu Y., Wang D., Feng R. (2018). Tribological properties of epoxy composite coatings reinforced with functionalized C-BN and H-BN nanofillers. Appl. Surf. Sci..

[7] Shahrokhi M., Mortazavi B., Berdiyorov G.R. (2017). New two-dimensional boron nitride allotropes with attractive electronic and optical properties. Solid State Commun..

[8] Tao X., Zhang L., Zheng X., Hao H., Wang X., Song L., Zeng Z., Guo H. (2018). *h*-BN/graphene van der Waals vertical heterostructure: A fully spin-polarized photocurrent generator. Nanoscale.

[9] Arutt C.N., Alles M.L., Liao W., Gong H., Davidson J.L., Schrimpf R.D., Reed R.A., Weller R.A., Bolotin K., Nicholl R. (2017). The study of radiation effects in emerging micro and nano electro mechanical systems (M and NEMS). Semicond. Sci. Technol..

[10] Garel J., Zhao C., Popovitz-Biro R., Golberg D., Wang W., Joselevich E. (2014). BCN nanotubes as highly sensitive torsional electromechanical transducers. Nano Lett..

[11] Bosak A., Serrano J., Krisch M., Watanabe K., Taniguchi T., Kanda H. (2006). Elasticity of hexagonal boron nitride: Inelastic X-ray scattering measurements. Phys. Rev. B Condens. Matter Mater. Phys..

[12] Song L., Ci L., Lu H., Sorokin P.B., Jin C., Ni J., Kvashnin A.G., Kvashnin D.G., Lou J., Yakobson B.I. (2010). Large scale growth and characterization of atomic hexagonal boron nitride layers. Nano Lett..

[13] Kim S.M., Hsu A., Park M.H., Chae S.H., Yun S.J., Lee J.S., Cho D.H., Fang W., Lee C., Palacios T. (2015). Synthesis of large-area multilayer hexagonal boron nitride for high material performance. Nat. Commun..

[14] Chopra N.G., Zettl A. (1998). Measurement of the elastic modulus of a multi-wall boron nitride nanotube. Solid State Commun..

[15] Suryavanshi A.P., Yu M.F., Wen J., Tang C., Bando Y. (2004). Elastic modulus and resonance behavior of boron nitride nanotubes. Appl. Phys. Lett..

[16] Eshkalak K.E., Sadeghzadeh S., Jalaly M. (2018). Mechanical properties of defective hybrid graphene-boron nitride nanosheets: A molecular dynamics study. Comput. Mater. Sci..

[17] Griebel M., Hamaekers J., Heber F. (2009). A molecular dynamics study on the impact of defects and functionalization on the young modulus of boron-nitride nanotubes. Comput. Mater. Sci..

[18] Li N., Ding N., Qu S., Liu L., Guo W., Wu C.M.L. (2017). Mechanical properties and failure behavior of hexagonal boron nitride sheets with nano-cracks. Comput. Mater. Sci..

[19] Wang H., Ding N., Zhao X., Wu C.M.L. (2018). Defective boron nitride nanotubes: Mechanical properties, electronic structures and failure behaviors. J. Phys. D Appl. Phys..

[20] Le M.Q. (2015). Prediction of young’s modulus of hexagonal monolayer sheets based on molecular mechanics. Int. J. Mech. Mater. Des..

[21] Mortazavi B., Rémond Y. (2012). Investigation of tensile response and thermal conductivity of boron-nitride nanosheets using molecular dynamics simulations. Phys. E Low-Dimens. Syst. Nanostruct..

[22] Wu J., Wang B., Wei Y., Yang R., Dresselhaus M. (2013). Mechanics and mechanically tunable band gap in single-layer hexagonal boron-nitride. Mater. Res. Lett..

[23] Mirnezhad M., Ansari R., Shahabodini A. (2013). Temperature effect on young’s modulus of boron nitride sheets. J. Therm. Stresses.

[24] Han T., Luo Y., Wang C. (2014). Effects of temperature and strain rate on the mechanical properties of hexagonal boron nitride nanosheets. J. Phys. D Appl. Phys..

[25] Oh E.S. (2010). Elastic properties of boron-nitride nanotubes through the continuum lattice approach. Mater. Lett..

[26] Hernández E., Goze C., Bernier P., Rubio A. (1999). Elastic properties of single-wall nanotubes. Appl. Phys. A Mater. Sci. Process..

[27] Ansari R., Mirnezhad M., Sahmani S. (2015). Prediction of chirality- and size-dependent elastic properties of single-walled boron nitride nanotubes based on an accurate molecular mechanics model. Superlattices Microstruct..

[28] Akdim B., Pachter R., Duan X., Adams W.W. (2003). Comparative theoretical study of single-wall carbon and boron-nitride nanotubes. Phys. Rev. B Condens. Matter Mater. Phys..

[29] Los J.H., Kroes J.M.H., Albe K., Gordillo R.M., Katsnelson M.I., Fasolino A. (2017). Extended tersoff potential for boron nitride: Energetics and elastic properties of pristine and defective *h*-BN. Phys. Rev. B.

[30] Thomas S., Ajith K.M., Chandra S., Valsakumar M.C. (2015). Temperature dependent structural properties and bending rigidity of pristine and defective hexagonal boron nitride. J. Phys. Condens. Matter.

[31] Boldrin L., Scarpa F., Chowdhury R., Adhikari S. (2011). Effective mechanical properties of hexagonal boron nitride nanosheets. Nanotechnology.

[32] Genoese A., Genoese A., Rizzi N.L., Salerno G. (2018). Force constants of BN, SiC, AlN and GaN sheets through discrete homogenization. Meccanica.

[33] Thomas S., Ajith K.M., Valsakumar M.C. (2016). Directional anisotropy, finite size effect and elastic properties of hexagonal boron nitride. J. Phys. Condens. Matter.

[34] Jiang L., Guo W. (2011). A molecular mechanics study on size-dependent elastic properties of single-walled boron nitride nanotubes. J. Mech. Phys. Solids.

[35] Kudin K.N., Scuseria G.E., Yakobson B.I. (2001). C_2_F, BN, and C nanoshell elasticity from ab initio computations. Phys. Rev. B Condens. Matter Mater. Phys..

[36] Peng Q., Ji W., De S. (2012). Mechanical properties of the hexagonal boron nitride monolayer: Ab initio study. Comput. Mater. Sci..

[37] Verma V., Jindal V.K., Dharamvir K. (2007). Elastic moduli of a boron nitride nanotube. Nanotechnology.

[38] Zhao S., Xue J. (2013). Mechanical properties of hybrid graphene and hexagonal boron nitride sheets as revealed by molecular dynamic simulations. J. Phys. D Appl. Phys..

[39] Le M.Q., Nguyen D.T. (2014). Atomistic simulations of pristine and defective hexagonal BN and SiC sheets under uniaxial tension. Mater. Sci. Eng. A.

[40] Vodenitcharova T., Zhang L.C. (2003). Effective wall thickness of a single-walled carbon nanotube. Phys. Rev. B.

[41] Wang C.Y., Zhang L.C. (2008). A critical assessment of the elastic properties and effective wall thickness of single-walled carbon nanotubes. Nanotechnology.

[42] Plimpton S. (1995). Fast parallel algorithms for short-range molecular dynamics. J. Comput. Phys..

[43] Tersoff J. (1988). New empirical approach for the structure and energy of covalent systems. Phys. Rev. B.

[44] Tersoff J. (1989). Modeling solid-state chemistry: Interatomic potentials for multicomponent systems. Phys. Rev. B.

[45] KinacI A., Haskins J.B., Sevik C., ÇaǧIn T. (2012). Thermal conductivity of BN-C nanostructures. Phys. Rev. B.

[46] Rajasekaran G., Kumar R., Parashar A. (2016). Tersoff potential with improved accuracy for simulating graphene in molecular dynamics environment. Mater. Res. Express.

[47] Mortazavi B., Cuniberti G. (2014). Mechanical properties of polycrystalline boron-nitride nanosheets. RSC Adv..

[48] Qi-Lin X., Zhen-Huan L., Xiao-Geng T. (2015). The defect-induced fracture behaviors of hexagonal boron-nitride monolayer nanosheets under uniaxial tension. J. Phys. D Appl. Phys..

[49] Wei A., Li Y., Datta D., Guo H., Lv Z. (2017). Mechanical properties of graphene grain boundary and hexagonal boron nitride lateral heterostructure with controlled domain size. Comput. Mater. Sci..

[50] Tabarraei A., Wang X. (2015). A molecular dynamics study of nanofracture in monolayer boron nitride. Mater. Sci. Eng. A.

[51] Wong C.H., Vijayaraghavan V. (2012). Nanomechanics of free form and water submerged single layer graphene sheet under axial tension by using molecular dynamics simulation. Mater. Sci. Eng. A.

[52] Zhang Y.Y., Xiang Y., Wang C.M. (2009). Buckling of defective carbon nanotubes. J. Appl. Phys..

[53] Yakobson B.I., Brabec C.J., Bernholc J. (1996). Nanomechanics of carbon tubes: Instabilities beyond linear response. Phys. Rev. Lett..

[54] Huang Y., Wu J., Hwang K.C. (2006). Thickness of graphene and single-wall carbon nanotubes. Phys. Rev. B.

[55] Batra R.C., Gupta S.S. (2008). Wall thickness and radial breathing modes of single-walled carbon nanotubes. J. Appl. Mech..

